# Biportal endoscopic decompression, debridement, and interbody fusion, combined with percutaneous screw fixation for lumbar brucellosis spondylitis

**DOI:** 10.3389/fsurg.2022.1024510

**Published:** 2023-01-06

**Authors:** Xiangbin Wang, Yubin Long, Yong Li, Yun Guo, Maiwulan Mansuerjiang, Zheng Tian, Aikebaier Younusi, Li Cao, Chong Wang

**Affiliations:** ^1^ Department of Orthopaedics, The First Affiliated Hospital of Xinjiang Medical University, Urumqi, China; ^2^ Department of Spinal Surgery, Hunan Shaoyang Central Hospital, Shaoyang, China

**Keywords:** biportal endoscopic technique, debridement, interbody fusion, lumbar brucellosis spondylitis, minimally invasive surgery

## Abstract

**Objective:**

This study aims to investigate the effectiveness and feasibility of biportal endoscopic decompression, debridement, and interbody fusion, combined with percutaneous screw fixation for lumbar brucellosis spondylitis (LBS).

**Methods:**

The data of 13 patients with LBS were retrospectively analyzed, who underwent biportal endoscopic decompression, debridement, and interbody fusion, combined with percutaneous screw fixation from May 2020 to June 2022. The patients’ clinical data, the duration of operation, the estimated blood loss (including postoperative drainage), and complications were recorded. Clinical outcomes include serum agglutination test (SAT) measures Brucella antibody titer, erythrocyte sedimentation rate (ESR), C-reactive protein (CRP), the visual analog scale (VAS) scores of low back and leg, Japanese Orthopaedic Association (JOA) score, Oswestry Disability Index (ODI), American Spinal Injury Association neurological classification, and lordotic angle were analyzed. All patients were assessed using the modified Macnab criteria at the final follow-up. The intervertebral bone graft fusion was assessed using the Bridwell grading criteria.

**Results:**

The mean operation duration was 177.31 ± 19.54 min, and the estimated blood loss was 176.15 ± 43.79 ml (including postoperative drainage was 41.15 ± 10.44 ml). The mean follow-up period was 13.92 ± 1.5 months. SAT showed that the antibody titers of 13 patients were normal 3 months after the operation and at the final follow-up. ESR and CRP levels returned to normal by the end of the 3-month follow-up. VAS scores of low back and leg, JOA score, and ODI significantly improved after the operation throughout the follow-up period (*P* < 0.05). Based on the modified Macnab criteria, 92.3% showed excellent to good outcomes. One patient had only a percutaneous screw internal fixation on the decompression side due to severe osteoporosis. One case suffered a superficial incision infection postoperatively that healed with dressing change and effective antibiotic treatment. Bony fusion was obtained in all patients at the last follow-up, including 12 cases with grade I and 1 case with grade II, with a fusion rate of 92.31%.

**Conclusion:**

Biportal endoscopic decompression, debridement, and interbody fusion, combined with percutaneous screw fixation is an effective, safe, and viable surgical procedure for the treatment of LBS.

## Introduction

Brucellosis is a zoonotic disease caused by Brucella that can affect multiple systems of the entire body, most commonly involving the musculoskeletal system (1). Osteoarticular infections occur mostly in the spine, and their prevalence has been reported in the literature to be approximately 6%–58% (2, 3), with the lumbar spine being the most frequent, followed by the thoracic and cervical spine (4, 5). The treatment of lumbar brucellosis spondylitis (LBS) remains controversial, and antibiotic chemotherapy is still considered to be the main treatment for the disease, usually with a good prognosis (6). Nevertheless, surgical intervention may be required for patients with progressive kyphotic deformity, neurological dysfunction, spinal instability, abscess formation, intractable low back pain, and failure to respond to conservative treatment (7, 8).

The biportal endoscopic technique is an emerging minimally invasive spine surgery that adopts two independent portals (viewing and working). An endoscope is placed in the viewing portal to monitor the surgical field, and instruments are placed in the working portal to perform the procedure. Several studies have shown excellent clinical results in the treatment of lumbar degenerative diseases with the biportal endoscopic technique (9–11). With the wide application of this technique in clinical practice recently, its surgical indications have gradually expanded and are not limited to lumbar degenerative diseases. Currently, some scholars have also attempted to apply this technique to treat spinal infectious lesions, such as epidural abscess (12), suppurative spondylitis (13), and spinal tuberculosis (14). To our knowledge, the biportal endoscopic technique for LBS has not been reported. Therefore, this study was conducted by retrospectively analyzing this group of cases and evaluating the clinical outcomes. This study aims to investigate the effectiveness and feasibility of biportal endoscopic decompression, debridement, and interbody fusion, combined with percutaneous screw fixation in the treatment of LBS and to summarize the surgical points and precautions.

## Materials and methods

### General information

**Figure 1:**
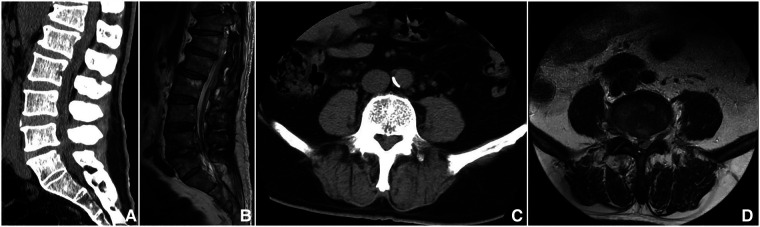
A 57-year-old male, whose complaint was intractable low back pain for 1 year and lower limb pain for 1 month. (**A,B**) Sagittal CT and MRI showed an epidural abscess compressing the thecal sac at L4–5. (**C,D**) Axial CT and MRI showed the epidural abscess.

The study protocol was approved by the Ethics Committee of the First Affiliated Hospital of Xinjiang Medical University and performed according to the Declaration of Helsinki. A total of 13 patients (10 males and 3 females) who were diagnosed with LBS who underwent biportal endoscopic decompression, debridement, and interbody fusion, combined with percutaneous screw fixation from May 2020 to June 2022 in our institution were included in this study (Table 1). The initial diagnosis of LBS was based on the presence of findings consistent with infection in the lumbar spine region on x-ray, computed tomography (CT), and magnetic resonance imaging (MRI) (15) (Figure 1 and Table 2), and confirmed diagnosis was done by positive blood culture, positive bacterial culture of a biopsy specimen, or serum agglutination test (SAT) revealing a titer of antibodies to Brucella of ≥1/160 (6). All patients were informed of all potential risks of the surgery and signed written consent preoperatively.

**Table 1:** Demographic characteristic of all patients.

Case No.	Gender	Age (years)	Level	Duration of symptoms (months)	SAT	Blood culture	Hospitalization (days)	Follow-up (months)
1	M	54	L2/3	3	1/320	N	8	17
2	M	37	L5/S1	9	1/640	N	10	16
3	F	56	L4/5	1	1/160	N	9	15
4	M	52	L5/S1	1	1/160	N	7	15
5	M	55	L3/4	4	1/320	N	6	13
6	M	59	L4/5	7	1/640	Y	7	13
7	F	44	L5/S1	4	1/320	N	12	12
8	M	59	L5/S1	3	1/320	Y	8	12
9	M	48	L5/S1	3	1/160	N	9	13
10	M	34	L4/5	7	1/640	Y	8	14
11	M	50	L3/4	12	1/640	N	9	14
12	M	57	L4/5	13	1/200	N	14	13
13	F	71	L1/2	4	1/160	N	19	14
Mean		52 ± 9.77		5.46 ± 3.89			9.69 ± 3.52	13.92 ± 1.5

M, male; F, female; SAT, serum agglutination test; N, negative blood culture; Y, positive blood culture.

**Table 2:** Radiological data of all patients.

Radiological studies	No. of patients (%)
X-ray	
Narrowing of disc space	7 (53.85)
Endplate lysis/sclerosis	5 (38.46)
Osteophyte formation	6 (46.15)
Destruction of vertebral body	4 (30.77)
Segmental instability	3 (23.08)
CT	
Narrowing of disc space	9 (69.23)
Endplate lysis/sclerosis	10 (76.92)
Osteophyte formation	7 (53.85)
Destruction of vertebral body	9 (69.23)
Spinal canal stenosis	10 (76.92)
Sequestrum	2 (15.38)
Segmental instability	4 (30.77)
MRI	
Disc involvement	4 (30.77)
Endplate involvement	11 (84.62)
Destruction of vertebral body	10 (76.92)
Epidural granulation tissue or abscess	12 (92.31)
Spinal canal stenosis	13 (100)
Segmental instability	3 (23.08)

CT, computed tomography; MRI, magnetic resonance imaging.

The inclusion criteria were as follows: (1) confirmed diagnosis of LBS combined with epidemiological history, clinical feature, laboratory, and imaging examinations; (2) the presence of intractable low back pain, severe or progressive neurological dysfunction, and imaging revealed massive epidural abscess; (3) ineffective conservative treatment (symptoms continued to worsen and/or infection could not be controlled); (4) the surgical approach was adopted with biportal endoscopic decompression, debridement, and interbody fusion, combined with percutaneous screw fixation; (5) postoperative follow-up ≥12 months. The exclusion criteria included (1) unclear diagnosis of LBS, or with other spinal infectious or neoplastic diseases; (2) lesions involving two or more segments; (3) patient unable to tolerate surgery; (4) those treated with other surgical modalities.

### Preoperative preparation

All patients received antibrucellosis chemotherapy orally in the form of doxycycline (200 mg/day) and rifampicin (600 mg/day) for at least 2 weeks preoperatively. Surgery was performed when the patient's temperature significantly decreased or was normal.

### Surgical methods

All procedures were performed under general anesthesia, with the patients in the prone position on a radiolucent table. These portals were checked under C-arm fluoroscopy guidance and marked, and then the skin of the surgical area was sterilized and the waterproof sterile surgical draping was used.

**Figure 2:**
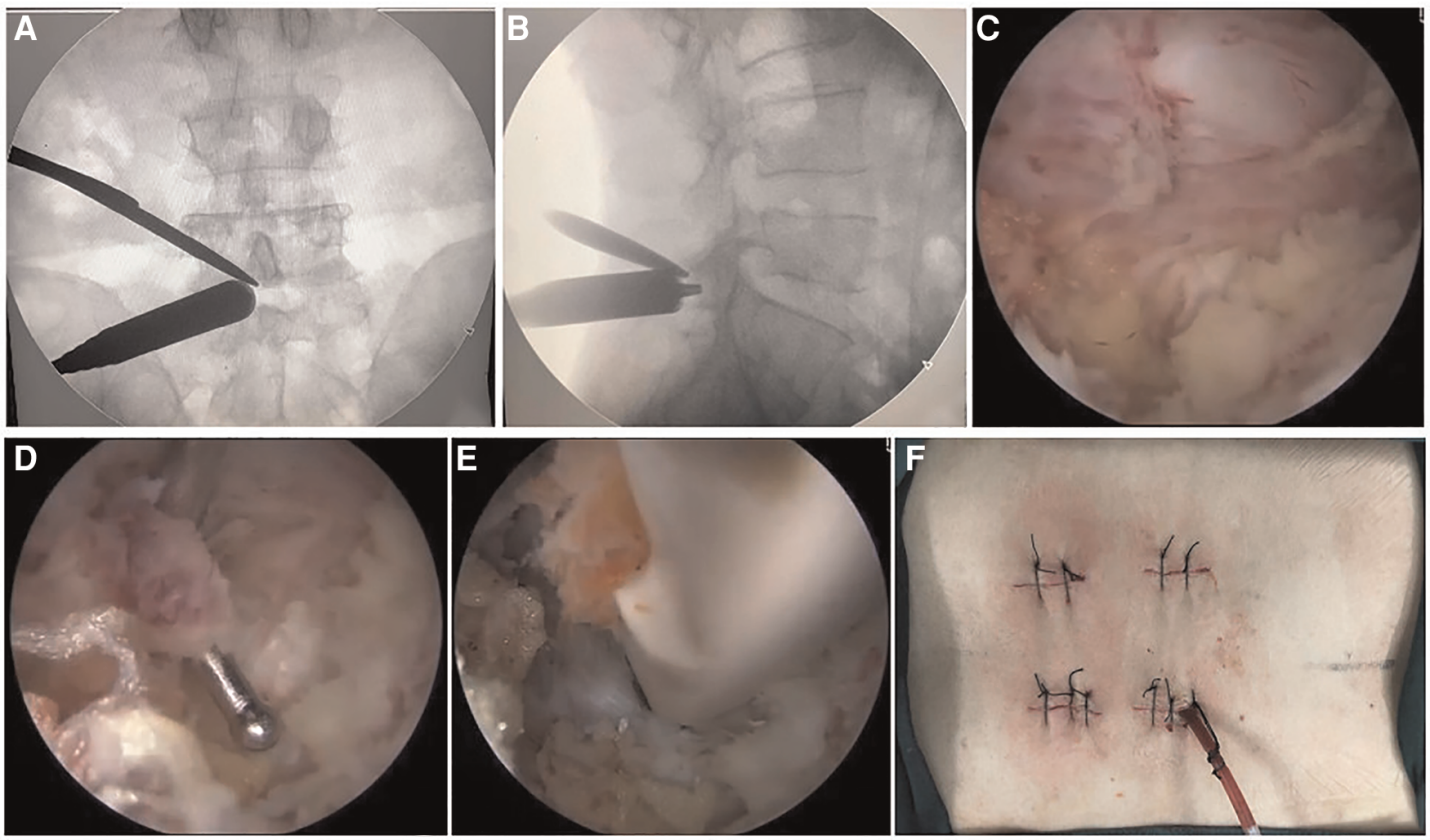
Intraoperative photographs during biportal endoscopic decompression, debridement, and interbody fusion, combined with percutaneous screw fixation. (**A,B**) The position of the two portals under the guidance of C-arm fluoroscopy. (**C**) The endoscopic image of light red inflammatory granulation tissue. (**D**) Endoscopic image of separated and exposed granulation tissue. (**E**) Endoscopic showed implantation of a cage filled with rifampin mixed with autologous bone. (**F**) Photograph of the incision after completion of surgery.

Two Kirschner needles were inserted into the marked portals used to precisely locate the intervertebral space in the anteroposterior and lateral views under fluoroscopy. Two portals were made for this procedure. The two holes were located 1 cm above and 1 cm below the center where the two needles’ junction points were located and placed close to the outer edge of the pedicle. The distance between the two channels may vary depending on the level and height of the patient, but the proximal channel is located approximately 2 cm above the distal channel. Two longitudinal incisions of about 1.5 cm were made to introduce the arthroscope and surgical instruments. For the left-sided approach, the cranial portal was used as the viewing portal to insert the arthroscope, and the caudal portal was used as the working portal to insert various instruments. The opposite was true on the right-sided approach. The fascia was incised perpendicular to the skin to prevent obstruction of water flow during the procedure. To facilitate the smooth flow of the flushing fluid, this can be achieved by extending the fascial incision or cutting across and manually placing a semitubular retractor. After making two small incisions in the fascia and skin, serial dilators were inserted under the guidance of C-arm fluoroscopy to create two holes. Then, a lamina dissector was used to dissect the lamina under the guidance of fluoroscopy (Figures 2A,B). The arthroscope system and instruments were inserted into two portals, and the irrigation fluid was drained naturally through the viewing portal toward the working portal without the assistance of a distractor or cannula.

When triangulation was established between the arthroscope and instrument, the soft tissue around the interlaminar space was cleared with an arthroscopic shaver. This revealed the lower margin of superior lamina, the upper margin of inferior lamina, the inferior articular process (IAP), and the facet joint. Ipsilateral laminectomy and facetectomy were performed first. An osteotome, Kerrison punch, and high-speed burr were used to remove the IAP, and part of the lower margin of superior lamina to the beginning of ligamentum flavum (LF) was exposed. Removed part of the upper margin of inferior lamina to the end of LF was exposed. Then, the apical and medial margins of the superior articular process (SAP) of the inferior vertebral body were removed to create a space between the traversing nerve root and the exiting nerve root. Concomitant contralateral decompression is performed for those with bilateral neurogenic symptoms or a high number of epidural abscesses. Local autologous bone harvested during the procedure was set aside for later use as interbody bone grafting. After completion of the ipsilateral decompression and contralateral decompression, as well as facetectomy. The LF overlying the dura and nerve roots were safely dissected, released, and completely removed intact using a rongeur and pulposus forceps for full exposure of the inflammatory lesion tissue, dura, and nerve release. After carefully dissecting the dura margin and nerve root, it could be safely protected with a specific retractor. It can be seen that the light red inflammatory granulation tissue compressed the dura and nerve root, carefully separated, and exposed the granulation tissue with a hook probe (Figures 2C,D). Enlarged vessels required a radiofrequency coagulator to coagulate. The inflammatory granulation tissue biopsy and removal were accomplished using various instruments such as pulposus forceps and Kerrison punch.

After protecting the dura and nerve roots with a special retractor, annulotomy was performed on the disc using a sharp knife. A group of reamers, a curette, and two pulposus forceps were used to perform the discectomy. Then, the lesioned nucleus pulposus was removed for a histopathological biopsy. The arthroscope was introduced into the intervertebral space to monitor the preparation of the endplate. The residual diseased tissue and nucleus pulposus were completely removed, the pus in the spinal canal and around the vertebral body was cleaned, the destroyed and sclerotic bone was curetted, and the cartilaginous endplate was removed cleanly with a curette to expose the subchondral bone until it seeped blood slightly. If the destruction of the endplate is obvious and the vertebral body is severely collapsed, only the removed bone is bitten into small pieces, mixed with rifampin, and implanted into the vertebral space. When the bones are insufficient, artificial bone or allogeneic bone can be taken. For those with intact upper and lower endplates and mild destruction, a cage can be implanted. A cage trial implant was inserted into the disc space to realign the height of the intervertebral disc while avoiding subchondral bone injury and to determine the size of the real cage. A special cannula was used to fill the anterior part of the disc space with rifampicin mixed with autologous bone collected from the lamina and facet owing to the concern of bone loss caused by continuous irrigation. After the nerve roots were protected with a retractor, the cage packed with rifampin autologous bone was carefully inserted under arthroscopic surveillance to avoid injury to the nerve root (Figure 2E). The cage was inserted deeper into the intervertebral space with the help of a hammer and demonstrated its position and size under fluoroscopy.

Finally, two percutaneous pedicle screws on the ipsilateral side were inserted through two previously described skin incisions, and two percutaneous pedicle screws were then contralaterally inserted after making two new skin incisions. Each of the screws was connected by the percutaneous insertion of a rod and the nuts were fixed. A drainage catheter was inserted to drain small bony debris or prevent epidural hematoma (Figure 2F).

### Postoperative management

**Figure 3:**
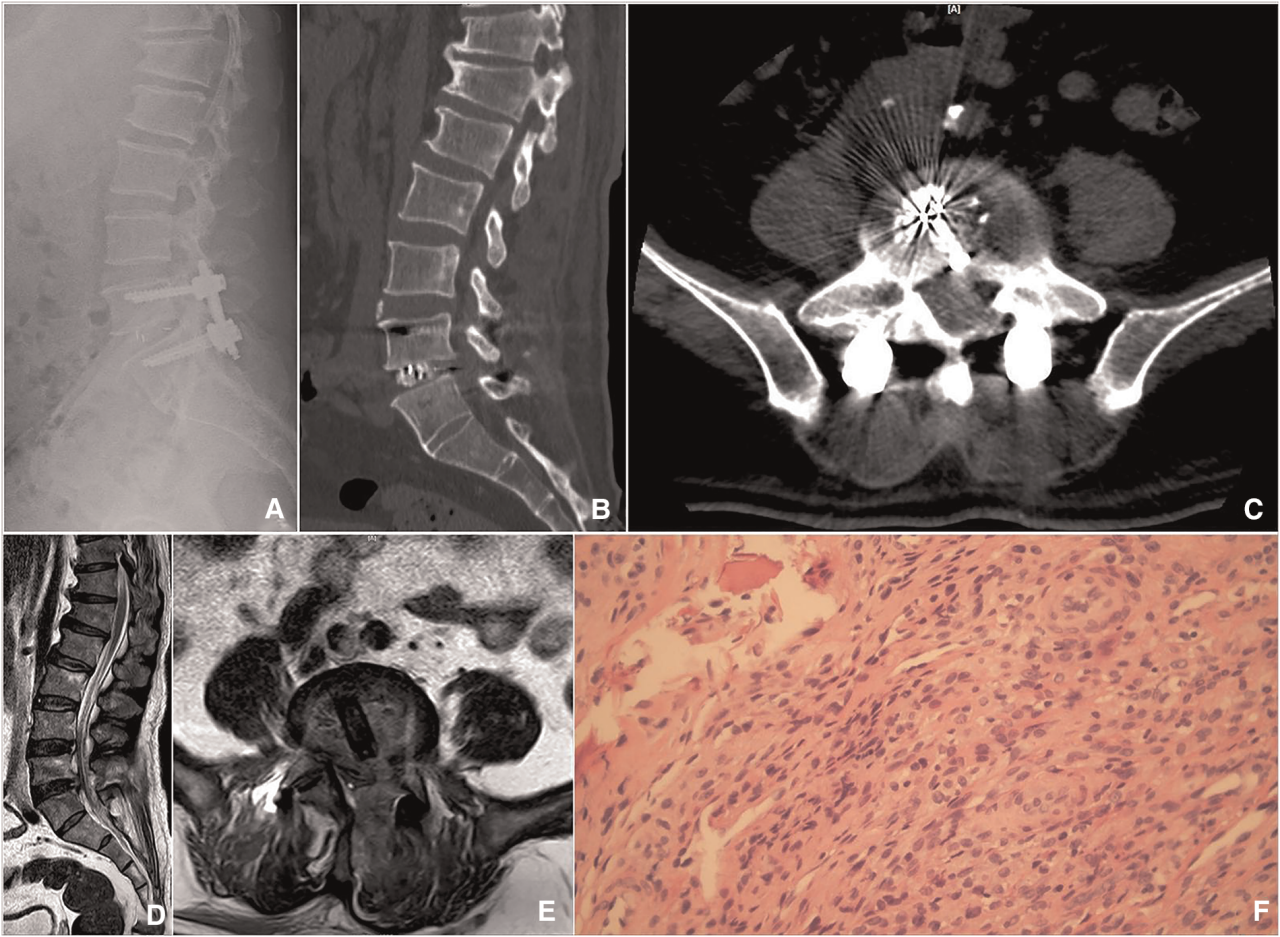
Postoperative imaging and pathological findings. (**A**) The lateral radiograph showed intervertebral bone grafting and instrumentation. (**B**) Sagittal CT showed that sufficient bone was planted. (**C**) Axial CT showed a good position of the Cage. (**D,E**) Sagittal and axial MRI showed sufficient decompression and abscess debridement. (**F**) Hematoxylin and eosin staining showed lymphocyte and monocyte infiltration in the samples biopsied.

Intravenous antibiotic (ceftriaxone, 2.0 g, Q12 h) was administered for 24 h postoperatively. Nonsteroidal anti-inflammatory drugs were used to reduce postoperative pain. The drainage tube was removed when the drainage flow was <30 ml/24 h. The patients were allowed to start walking with a lumbar brace 1 day postoperatively. All patients received the WHO-recommended oral regimen, consisting of doxycycline (200 mg/day) and rifampicin (600 mg/day) for a minimum of 3 months after the operation. X-ray and CT were performed on all patients before discharge to evaluate the location of the graft and instrumentation (Figures 3A–C). The decompression and abscess clear were assessed by sagittal and axial MRI (Figures 3D,E). Lumbar brace protection continued for 3 months.

### Main observation indicators

The mean operative time, estimated blood loss (including postoperative drainage), and complications were recorded. Clinical outcomes include SAT measures Brucella antibody titer, erythrocyte sedimentation rate (ESR), C-reactive protein (CRP), the visual analog scale (VAS) scores of low back and leg, Japanese Orthopaedic Association (JOA) score, Oswestry Disability Index (ODI), American Spinal Injury Association (ASIA) neurological classification, and lordotic angle. All patients were examined clinically and radiologically at 1, 3, 6, and 12 months postoperatively and were assessed using the modified Macnab criteria at the final follow-up. The intervertebral bone graft fusion was assessed using the Bridwell grading criteria (16). When there was uncertainty on x-ray, further evaluation was done by CT.

### Statistical methods

The data were statistically analyzed by using SPSS 26.0 software. The measurement data are expressed as the mean ± standard deviation (SD), significant differences in quantitative scores (VAS, JOA, and ODI) were determined using repeated-measures analysis of variance, and Student's *t*-test was used to evaluate changes in lordotic angle and laboratory (ESR and CRP). Any discrepancy in normal distribution was analyzed using the rank sum test. *P* < 0.05 was considered to be statistically significant.

## Results

### General results and complications

A total of 13 patients [10 males and 3 females, mean age ( ± SD) was 52 ± 9.77 years] who met the criteria were included in our study. The mean length of hospital stay was 9.69 ± 3.52 days, and the average follow-up time was 13.92 ± 1.5 months. The infectious levels included L1–2 in one patient, L2–3 in one patient, L3–4 in two patients, L4–5 in four patients, and L5–S1 in five patients (Table 1). All the patients completed the operation successfully. The mean operation duration was 177.31 ± 19.54 min, the estimated blood loss was 176.15 ± 43.79 ml (including postoperative drainage was 41.15 ± 10.44 ml), the average postoperative hospitalization time was 5 ± 2.31 days, and the time to ambulation was 1–2 days (Table 3).

**Table 3:** Results related to surgery.

Variable	Value
Operative time (minutes)	177.31 ± 19.54
Estimated blood loss (ml)	176.15 ± 43.79
Postoperative drainage (ml)	41.15 ± 10.44
Time to ambulation (days)	1–2
Postoperative hospitalization time (days)	5 ± 2.31

One patient underwent percutaneous screw internal fixation on the decompression side only due to severe osteoporosis. The time of bed rest and wearing a lumbar brace were prolonged after the operation and were treated with regular oral medication against osteoporosis. A superficial incision infection, which may be caused by the poor general condition of the patient, was observed in one patient postoperatively that healed with dressing change and intravenous antibiotic treatment. No perioperative complications related to decompression or instrumentation.

### Symptom function

The clinical symptoms are summarized in Table 4. All cases had significant improvement in constitutional symptoms and lower back pain after the procedure. The VAS scores of lower back and leg, JOA score, and ODI significantly improved before discharge, 1, 3, 6 months, and the last follow-up compared with those before the operation, and the differences were statistically significant (*P* < 0.05) (Table 5). Based on the modified Macnab criteria, the outcomes were excellent in 10 cases (76.92%), good in 2 cases (15.38%), acceptable in 1 case (7.7%), and none of the patients showed poor outcomes. 92.3% showed excellent to good outcomes. In eight cases ASIA was E, and in five cases ASIA was D preoperatively. Two patients recovered to E before discharge and three patients improved to E at the last follow-up (Table 4).

**Table 4:** Clinical features of all patients.

Clinical features	No. of patients (%)
Spinal symptoms	
Low back pain	13 (100)
Leg pain	11 (84.62)
Constitutional symptoms	
Fever	3 (23.08)
Sweating	4 (30.77)
Loss of appetite or weakness or fatigue	13 (100)
Weight loss	7 (53.85)
Arthralgia	2 (15.38)
ASIA classification	
D	5 (38.46)
E	8 (61.54)

ASIA, American Spinal Injury Association.

**Table 5:** Clinical outcomes (VAS, JOA, and ODI) of pre- and postsurgery.

Date	VAS score of low back	VAS score of leg	JOA score	ODI score (%)
Preoperative	5.85 ± 1.28	3.69 ± 2.02	13.46 ± 3.18	55.57 ± 10.99
Before discharge	3.15 ± 0.8^a^	1.92 ± 1.19^a^	—	—
Post				
1 month	1.92 ± 0.64^a^	1.31 ± 0.75^a^	20.85 ± 2.91^a^	37.09 ± 9.99^a^
3 months	1.38 ± 0.51^a^	1.08 ± 0.64^a^	24.77 ± 1.92^a^	26.54 ± 6.96^a^
6 months	0.85 ± 0.8^a^	0.69 ± 0.48^a^	25.92 ± 1.04^a^	10.63 ± 2.91^a^
Final follow-up	0.38 ± 0.51^a^	0.23 ± 0.44^a^	27.08 ± 0.95^a^	6.14 ± 3.38^a^
*P* value	*P* < 0.05	*P* < 0.05	*P* < 0.05	*P* < 0.05

VAS, visual analog scales; JOA, Japanese Orthopaedic Association; ODI, Oswestry Disability Index.

^a^ Significantly different from the preoperative value (P < 0.05).

### Laboratory indicators

Postoperatively all histopathological biopsies showed noncaseating granulomatous inflammation, with a large number of lymphocytes and monocytes (Figure 3F), all consistent with the diagnosis of brucellosis spondylitis. SAT showed that the antibody titers of 13 patients were normal 3 months after the operation and at the final follow-up. The preoperative, before discharge, postoperative 1, 3, 6 months, and the last follow-up ESR were 38.69 ± 18.98, 36.23 ± 11.39, 24.85 ± 9.17, 8.77 ± 3.72, 8.46 ± 2.73, and 5.92 ± 2.81 mm/h, respectively. However, there was no significant difference between preoperative ESR and before discharge (from 38.69 ± 18.98 to 36.23 ± 11.39 mm/h, *t* = 1.413, *P* > 0.05). Compared with preoperative ESR, it significantly decreased at postoperative 1 month (from 38.69 ± 18.98 to 24.85 ± 9.17, *t* = 3.705, *P* < 0.05). Compared with preoperative ESR, it significantly decreased at postoperative 3 months (from 38.69 ± 18.98 to 8.77 ± 3.72, *t* = 7.630, *P* < 0.05). Compared with preoperative ESR, it significantly decreased at postoperative 6 months (from 38.69 ± 18.98 to 8.46 ± 2.73, *t* = 7.787, *P* < 0.05). Compared with preoperative ESR, it significantly decreased at the last follow-up (from 38.69 ± 18.98 to 5.92 ± 2.81, *t* = 6.158, *P* < 0.05). The preoperative, before discharge, postoperative 1, 3, 6 months, and the last follow-up CRP were 26.82 ± 19.87, 29.56 ± 14.32, 13.72 ± 6.03, 5.45 ± 1.84, 5.13 ± 1.75, and 4.25 ± 1.91 mg/L, respectively. However, there was no significant difference between preoperative CRP and before discharge (from 26.82 ± 19.87 to 29.56 ± 14.32 mg/L, *t* = −0.404, *P* > 0.05). Compared with preoperative CRP, it significantly decreased at postoperative 1 month (from 26.82 ± 19.87 to 13.72 ± 6.03, *t* = 2.275, *P* < 0.05). Compared with preoperative CRP, it significantly decreased at postoperative 3 months (from 26.82 ± 19.87 to 5.45 ± 1.84, *t* = 3.862, *P* < 0.05). Compared with preoperative CRP, it significantly decreased at postoperative 6 months (from 26.82 ± 19.87 to 5.13 ± 1.75, *t* = 3.921, *P* < 0.05). Compared with preoperative CRP, it significantly decreased at the last follow-up (from 26.82 ± 19.87 to 4.25 ± 1.91, *t* = 4.077, *P* < 0.05).

### Radiographic results

**Figure 4:**
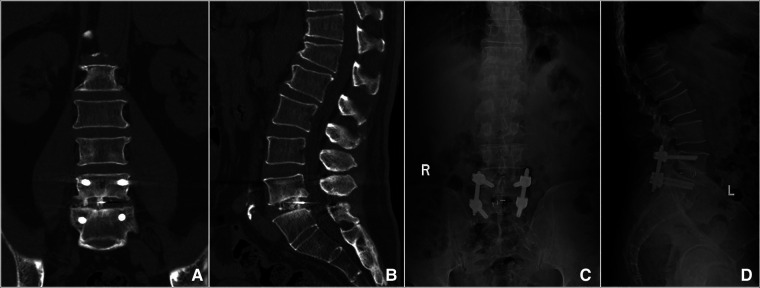
Imaging findings during postoperative follow-up. (**A,B**) Coronal and sagittal CT showed that the cage was well positioned and high-density bone fusion between vertebral bodies at 6 months postoperatively. (**C,D**) A 14-month postoperative x-ray showed bony fusion and the instrumentation was in a good position.

The preoperative, before discharge, and the final follow-up lordotic angle were 47.18 ± 6.88°, 40.83 ± 6.71°, and 42.26 ± 6.92°, respectively. Compared with preoperative lordotic angle, it significantly decreased before discharge (from 47.18 ± 6.88° to 40.83 ± 6.71°, *t* = 2.384, *P* < 0.05). However, there was no significant difference between the preoperative lordotic angle and the final follow-up (from 47.18 ± 6.88° to 42.26 ± 6.92°, *t* = 1.819, *P* > 0.05). Lordotic angle decreased postoperatively and there was no significant loss of angle at the last follow-up. The x-ray or CT (Figures 4A,B) at 6 months after the operation showed that seven cases (53.85%) had a segmental fusion, five cases (38.46%) had fusion trends but not fused, and one case (7.69%) showed no segmental fusion, in which bony fusion was obtained in all patients at the last follow-up (Figures 4C,D), including 12 cases with grade I and 1 case with grade II, with a fusion rate was 92.31%. Lumbar flexion and extension radiographs as well as CT were performed on this patient, and no pseudarthrosis was found. No loosening or fracture of the internal fixation occurred in all patients.

## Discussion

The incidence rate of brucellosis is very high, with more than half a million new cases annually, which has become a world public health problem and has brought a huge burden to society and the economy, especially in underdeveloped regions (17). Osteoarticular infections are one of the common manifestations of brucellosis, especially the lumbar spine is the predilection site of brucellosis, accounting for 6%–12% of all sites, which is the foremost cause of the debilitating and disabling complications (18). Combinations of antimicrobial chemotherapy remain the mainstay of treatment for LBS and are curative in most cases with conservative drug therapy (19), but residual kyphosis and spinal instability are found in certain patients at the end of treatment. The diagnosis and treatment of the disease pose great challenges to physicians. Due to delayed diagnosis and treatment, some patients suffer from neurological dysfunction by compressing effects from inflammatory granulation tissue or abscesses, intractable or progressive low back pain due to spinal instability, or massive paravertebral abscess formation; antibiotic therapy is also ineffective. For such patients, surgical treatment is frequently imperative (7, 8). Nevertheless, there is no consensus on the optimal surgical approach to the disease and the role of surgical intervention. In this study, the main goals of the surgery were to completely remove the infected lesion, relieve or eliminate pain, relieve compression, improve function, rebuild spinal stability, and restore normal spinal sequence.

There are few reports in the literature related to the surgical treatment of LBS, and the main surgical approaches include anterior debridement, traditional posterior opening surgery, and combined anterior and posterior approaches. Nontuberculous spinal infections were successfully treated through anterior debridement, fusion, and fixation by Redfern et al. (20). Anterior debridement, interbody fusion, and internal fixation were performed for LBS in 2018 by Yin et al. (21), with excellent clinical outcomes. Anatomically, brucellosis spondylitis usually begins at the superior endplate of the anterior margin due to the rich blood supply in this region (15). Thus, anterior surgery achieves adequate debridement and neurological decompression without compromising posterior spinal stability. However, there are still many shortcomings in the anterior approach. The anterior approach takes longer and may have complications such as vascular injury, ureteral injury, postoperative ileus, and bone graft failure compared with posterior open surgery (22–24). For cases with inflammatory granulation tissue or abscesses in the spinal canal, posterior open surgery can be directly performed to remove the compression, and with spinal instability or kyphotic deformity, pedicle screw internal fixation can also be performed to maintain or reconstruct spinal stability, correct deformity, and promote bone graft fusion, thus effectively treating LBS. Although conventional posterior open surgery compensates for the anterior approach, its disruption of the posterior musculoligamentous structures can lead to complications such as chronic low back pain and muscle atrophy after the procedure (25).

The distribution of abscess in brucellosis spondylitis is relatively limited, mainly involving the endplate and intervertebral space of the affected segment, and most of the bone destruction is dominated by sclerotic bone, unlike tuberculous spondylitis. The complete removal of the diseased tissue should not be overemphasized when debridement of the lesion is performed, as this may cause loss of residual bone and result in spinal instability. For this reason, biportal endoscopic decompression, debridement, and interbody fusion, combined with percutaneous screw fixation was adopted by us for patients with LBS, which has less injury to the posterior musculoligamentous and bony structures. Decompression and debridement under endoscopic surveillance are safer and more efficient, which can ensure adequate decompression and effective lesion removal while preserving more normal musculoligamentous and bony structures, thereby reducing the complications such as postoperative low back pain, muscle atrophy, and spinal instability. Moreover, percutaneous screw fixation can effectively maintain or reconstruct spinal stability and promote bony healing. The patients with epidural abscesses were successfully treated using the biportal endoscopic technique by Kang et al. (12). The unilateral biportal endoscopic discectomy and debridement were performed on salmonella spondylitis with epidural abscess by Hsu et al. (13), with an excellent outcome. In 2021, Kim et al. (14) applied the first biportal endoscopic debridement and percutaneous screw fixation technique for spinal tuberculosis. A total of 13 patients with LBS included in this study achieved satisfactory clinical outcomes and met the criteria for clinical cure after surgery. Through the above series of literature reports and the results of our study, it is feasible to treat LBS with biportal endoscopic decompression, debridement, and interbody fusion, combined with percutaneous screw fixation under strict control of the indications for the procedure.

With the widespread clinical application of biportal endoscopic technique in recent years, its surgical indications have been extended from lumbar disc herniation (9) and lumbar spinal stenosis (26) to lumbar interbody fusion (27), spinal infectious diseases (12–14), and even epidural tumor (28), and the therapeutic effect is comparable to that of convention open surgery. Decompression and debridement were performed by the biportal endoscopic technique under visualization resulting in more adequate decompression and lesion removal complete; handling the intervertebral space and bone graft fusion under endoscopic surveillance makes endplate preparation more complete and implantation of bone graft and cage safer. The technique has the advantages of clear vision, large working space, and freedom of operation and can be decompressed using traditional spinal surgical instruments. It combines the features of endoscopic and open surgery and truly embodies the minimally invasive concept of endoscopic operation.

The surgical points and precautions of biportal endoscopic decompression, debridement, and interbody fusion, combined with percutaneous screw fixation were summarized as follows: (1) the order of decompression: after determination of the interlaminar space, first decompressed the bony structures, followed by the LF. After the IAP and the inferior margin of superior lamina were removed, then the upper edge of inferior lamina was resected, and subsequently, the medial edge and apex of the SAP were removed. Unilateral laminectomy and bilateral decompression should be performed for bilateral neurogenic symptoms or massive abscesses in the spinal canal. (2) When spinal infectious diseases are treated, it is recommended that the ipsilateral LF be preserved first, which reduces the risk of injury to the dura and ipsilateral nerve root from surgical instruments during contralateral decompression. (3) In patients with LBS, there is significant vascular proliferation, rich blood supply, and easy bleeding. If the ligamentum flavum is removed first, the surgical field is blurred due to hemorrhage, which increases the risk of nerve injury. Especially for epidural abscess or diseased tissue compressing the dura mater and nerve roots, dura mater dilatation significantly causes difficulty in “overtopping” and increased the risk of injury. (4) In the case of LBS, there may be inflammatory tissue adhering LF to the dura densely. In such cases, frequent gentle tractions of LF from the dura with punch and pituitary forceps are helpful for spontaneous detachment and gentle separation of the inflammatory tissue from the dura using a separator. The careful insertion of a blunt hook over the dura will prevent tears in the dura, which leads to adhesiolysis by saline irrigation into the epidural space between the inflammatory tissue, dura, and the overlying LF. (5) The rifampicin mixed with autologous bone implanted can provide an effective local anti-infection effect. Careful hemostasis and clear visualization should be maintained intraoperatively, and it is not advisable to maintain clear visualization by increasing water pressure to prevent the development of spinal cord hypertension syndrome.

The indications for this procedure are similar to those for conventional open surgery: (1) severe disc destruction or vertebral infection resulting in intractable low back pain that cannot be relieved by medication treatment. (2) Severe or progressive neurological dysfunction due to compression of the spinal cord or cauda equina and nerve roots by inflammatory granulation tissue in the spinal canal or epidural abscesses. (3) Spinal instability due to vertebral was destroyed. (4) Drug antibrucellosis therapy was ineffective. Limitations of this operation: (1) the anterior column (anterior longitudinal ligament, anterior two-thirds of the vertebral body, and fibrous ring) was severely destroyed, or massive abscess formation at the anterior margin required for anterior debridement and interbody fusion fixation *via* a retroperitoneal approach, or the formation of a massive paravertebral abscess. (2) Incomplete debridement and decompression may occur because of unclear vision. (3) The retroperitoneum may rupture and enter the abdominal cavity leading to peritoneal effusion and infection due to the continuous flow of large amounts of irrigation fluid (29).

Posterior debridement and decompression were performed by biportal endoscopic technique. Surgeons are concerned that they could cause intraspinal and central nervous system infections. Chen et al. (30) reported 24 cases of posterior debridement, bone grafting, and internal fixation for brucellosis spondylitis with significant improvement in VAS scores and neurological function after surgery, and no recurrent cases were found during follow-up. Sixty-two patients with LBS treated with posterior debridement and bone grafting combined with internal fixation were reported by another study (31), and all cases were clinically cured at the final follow-up. Surgeons have also expressed concern about the possibility of an increased risk of recurrence due to the spread of the lesion by flowing saline during resection of the lesion. However, it is worth noting that the biportal endoscopic technique for spinal infectious diseases has been previously reported in the literature (12–14) and achieved excellent outcomes. Furthermore, the use of the percutaneous endoscopic technique for spinal infectious diseases such as pyogenic spondylitis and spinal tuberculosis has also been reported with good results (32, 33). In addition, some scholars have expressed concern about the risk of infection with the use of implants because this may decrease the effectiveness of antibiotics while increasing bacterial adherence and glycocalyx formation. Notably, the adherence properties of *Staphylococcus epidermidis* to stainless steel were investigated by Oga et al. (34) and found that the bacteria colonized the rods in large numbers. Nevertheless, Chang and Merritt (35) concluded that titanium is less prone to bacterial colonization than polymethyl-methacrylate and stainless steel materials. The safety and efficacy of the titanium alloy screw-rod system in the treatment of spinal infectious diseases were confirmed by relevant studies, but it is necessary to perform effective debridement for the infected lesions, as well as take antibacterial drugs regularly and fully after the operation (35–37). In our study, the results were consistent with the aforementioned literature, with no cases of intraspinal and central nervous system infection, no recurrence found, and no instrumentation-related complications during follow-up, which may be related to regular antibrucellosis treatment before and after the procedure, continuous saline irrigation intraoperatively, and administered intravenous antibiotics perioperatively. Furthermore, local antibiotics and percutaneous screw fixation play an important role in the treatment of spinal infection, which is conducive to inhibiting infection, providing a relatively stable internal environment, and preventing recurrence (38).

Interbody fusion using autologous bone graft has been shown to be good practice for spinal infections. Several academics have apprehension about the use of cages in the treatment of spinal infections. For these reasons, Zhao et al. (39) adopted polyetheretherketone (PEEK) cages in combination with one-stage posterior debridement and instrumentation for 61 cases with LBS in 2020, which all patients had a successful outcome in terms of clinical and radiological findings after the operation, particularly no recurrence was detected at the 12-month follow-up. In this study, the reasons why we chose the cage instead of the autologous ilium to promote fusion are as follows: (1) Most of the bone destruction in brucellosis spondylitis is predominantly sclerotic bone, with the majority of patients having intact upper and lower endplates and lesser destruction (15). A total of 13 patients included in this study have intact endplates. (2) Ilium grafts have complications at the donor site, such as high levels of infection and hematoma, as well as limited iliac bone material in elderly patients (40), which is more invasive, bleeds more, takes longer to operate, and may result in prolonged bed rest due to intractable pain in the bone extraction area after surgery (41). (3) Autologous bone implants alone may have insufficient support, prolonged fusion time, and resorption of a small amount of bone, which may result in weaker recovery of the intervertebral space and foraminal height than cage fusion (42). (4) A cage as a carrier for bone grafting, based on the “brace-compression” principle, has a strong support effect, better biomechanical stability, facilitates early fusion, maintains the height of the spinal space, reduces the possibility of other pathologies due to pressure changes in the spine, and reduces the fatigue stress on the posterior nail bar system (43). (5) Because of the risk of bacterial biofilm formation, insertion of implants in infected areas is generally contraindicated. However, for spinal tuberculosis, some authors deem that there is less risk of such bond formation as *Mycobacterium tuberculosis* proliferated slowly with minimal glycocalyx slime production and existed in a planktonic form, which responded well to chemotherapy (44). Based on the above reports in the literature and considering that brucellosis is less aggressive than spinal tuberculosis (45), we tried to apply autogenous bone with a cage for intervertebral fusion and obtained a satisfactory fusion rate after the surgery, and no graft-related complications, such as cage subsidence and infection. Thus, interbody fusion with cage plus autologous bone is safe and feasible for those with intact endplates in LBS, and it has been reported that local bone graft with a cage is as beneficial as that without a cage (46).

In our research, 13 patients with LBS had significant relief of low back pain and radiating leg pain after the surgery, and VAS scores of low back and leg, JOA score, and ODI were significantly improved compared with those before the surgery, which further improved with time. The modified Macnab criteria showed excellent to good outcomes of 92.3%. Patients with neurological dysfunction improved after the operation, and all returned to normal at the final follow-up. The reason for this is that, on the one hand, biportal endoscopic decompression and debridement can relieve the compression of the spinal cord or cauda equina and nerve roots by inflammatory granulation tissue or abscess, and large amounts of saline continuous intraoperative flushing can remove most of the inflammatory factors, pus, and pathogenic bacteria and discharge them in time, eliminating the stimulation of inflammatory pain-causing factors, and effectively reducing intervertebral space pressure, thus significantly reducing pain. On the other hand, interbody fusion and percutaneous screw fixation can reconstruct or maintain spinal stability and improve severe low back pain caused by spinal instability owing to the lesion invading the vertebral body. Even if there was no significant improvement in ESR before discharge compared to before operation and CRP was elevated than before the procedure, the number of patients with ESR and CRP returning to normal increased continuously as time progressed. Possible reasons for this were analyzed were inflammation stimulation due to surgical trauma and the normal course of the disease. Moreover, though some patients in this study had spinal instability because of vertebral destruction, most of them had no significant kyphotic deformity. Hence, this may explain why the postoperative lordosis angle did not change significantly from the preoperative one. All patients showed bony fusion by reexamination of x-ray or CT at the final follow-up, including 12 cases with grade I and 1 case with grade II, with a fusion rate was 92.31%. The authors considered that the patient's fusion was grade II might be related to the following reasons: (1) the patient has severe osteoporosis; (2) the PEEK cage has a smooth surface and low bioactivity, which affects bone conductivity (47); (3) local use of rifampicin may inhibit the growth and mineralization of osteoblasts (48, 49); (4) prolonged and continuous saline irrigation has an effect on bone healing, but the exact mechanism is not known. Flexion and extension radiographs as well as CT were performed on this patient, and no pseudoarthrosis formation was detected, and no loosening or fracture of the internal fixation occurred in all patients. One case had percutaneous screw fixation on the decompression side only as a result of severe osteoporosis and was treated with postoperative antiosteoporosis medication. One patient developed a superficial incision infection postoperatively, which was considered due to the patient's obesity and history of diabetes, which healed with dressing change and effective antibiotic treatment.

Our research has some limitations such as it being a small sample size retrospective study with a lack of control groups, and to our knowledge, the biportal endoscopic technique was first utilized for LBS in this study, and its safety and efficacy need to be confirmed by the results of more clinical studies. Additionally, the follow-up period was short, and further evidence is needed for the certainty of long-term efficacy and the impact on spinal stability. Nonetheless, the symptoms, signs, laboratory, and imaging results of the patients included in this study were significantly improved postoperatively, indicating that biportal endoscopic decompression, debridement, and interbody fusion, combined with percutaneous screw fixation is feasible and effective in the treatment of LBS.

## Conclusion

Pharmacological antimicrobial chemotherapy is the basis of treatment for LBS, and surgery is inevitable when the patient has intractable low back pain, severe or progressive neurological dysfunction, and spinal instability and conservative treatment is ineffective. Biportal endoscopic decompression, debridement, and interbody fusion, combined with percutaneous screw fixation is an effective, safe, and viable surgical procedure that should be considered a choice for the treatment of LBS.

## Data Availability

The raw data supporting the conclusions of this article will be made available by the authors, without undue reservation.
